# Current Research Trends in Glioblastoma: Focus on Receptor Tyrosine Kinases

**DOI:** 10.3390/ijms26083503

**Published:** 2025-04-09

**Authors:** Edmond Nicolae Barcan, Carmen Duta, Georgiana Adeline Staicu, Stefan Alexandru Artene, Oana Alexandru, Alexandra Costachi, Andreea Silvia Pirvu, Daniela Elise Tache, Irina Stoian, Stefana Oana Popescu, Ligia Gabriela Tataranu, Anica Dricu

**Affiliations:** 1 Department of Biochemistry, University of Medicine and Pharmacy of Craiova, Petru Rares 2, 200349 Craiova, Romania; edmond.barcan@gmail.com (E.N.B.); adstaicu@gmail.com (G.A.S.); stefan.artene@yahoo.com (S.A.A.); andreeaneamtu1989@yahoo.com (A.S.P.); danielaelisetache@gmail.com (D.E.T.); 2 Department of Biochemistry, Carol Davila University of Medicine and Pharmacy, 020022 Bucharest, Romania; carmen.duta@umfcd.ro (C.D.); irina.stoian@umfcd.ro (I.S.); anica.dricu@umfcd.ro (A.D.); 3 Department of Neurology, University of Medicine and Pharmacy of Craiova, Petru Rares 2, 200349 Craiova, Romania; oanale@hotmail.com; 4 Faculty of Pharmacy, University of Medicine and Pharmacy of Craiova, Petru Rares 2, 200349 Craiova, Romania; alexandra.costachi@yahoo.com; 5 Department of Neurosurgery, Clinical Emergency Hospital “Bagdasar-Arseni”, Soseaua Berceni 12, 041915 Bucharest, Romania; 6 Department of Neurosurgery, Carol Davila University of Medicine and Pharmacy, 020021 Bucharest, Romania

**Keywords:** glioblastoma, receptor tyrosine kinases, -omics sciences, molecular diagnostics

## Abstract

Glioblastoma (GBM) is an aggressive brain tumor characterized by molecular complexity and resistance to conventional treatments, including surgery, radiation, and chemotherapy. Despite these challenges, advancements in receptor tyrosine kinase (RTK) research, combined with multi-omics approaches, hold promise for improving patient outcomes and survivability. RTKs are central to GBM progression, influencing cell proliferation, survival, and angiogenesis. However, the complexity of RTK signaling necessitates a broader, integrative perspective, which has been enabled by the emergence of -omics sciences. Multi-omics technologies—including genomics, transcriptomics, proteomics, and metabolomics—offer unprecedented insights into the molecular landscape of GBM and its RTK-driven pathways. Genomic studies have revealed mutations and amplifications in RTK-related genes, while transcriptomics has uncovered alterations in gene expression patterns, providing a clearer picture of how these aberrations drive tumor behavior. Proteomics has further delineated changes in protein expression and post-translational modifications linked to RTK signaling, highlighting novel therapeutic targets. Metabolomics complements these findings by identifying RTK-associated metabolic reprogramming, such as shifts in glycolysis and lipid metabolism, which sustain tumor growth and therapy resistance. The integration of these multi-omics layers enables a comprehensive understanding of RTK biology in GBM. For example, studies have linked metabolic alterations with RTK activity, offering new biomarkers for tumor classification and therapeutic targeting. Additionally, single-cell transcriptomics has unveiled intratumoral heterogeneity, a critical factor in therapy resistance. This article highlights the transformative potential of multi-omics in unraveling the complexity of RTK signaling in GBM. By combining these approaches, researchers are paving the way for precision medicine strategies that may significantly enhance diagnostic accuracy and treatment efficacy, providing new hope for patients facing this devastating disease.

## 1. Introduction

Glioblastoma (GBM) is the most prevalent primary malignant brain tumor, accounting for 16% of all primary brain and central nervous system tumors. The average incidence rate, adjusted for age, is 3.2 per 100,000 people [1]. GBM is classified as grade IV malignant glioma by the World Health Organisation (WHO) [2]. Few patients survive GBM in the long term. The survival rate of 10 years or more in the population of GBM cases is less than 1%. An increased likelihood of achieving 10-year survival is associated with younger age at diagnosis [1]. Due to having a very complex genetic basis, GBM is potentiated by dysregulation of a multitude of signaling pathways. This provided a huge number of opportunities for more targeted therapies in GBM [3,4].

At the moment, the standard treatment includes maximal safe surgical resection, followed by concurrent radiation and temozolomide (TMZ), an oral alkylating chemotherapy agent, and then adjuvant chemotherapy with TMZ [5,6].

Achieving extensive and complete surgical resection is challenging due to the invasive nature of these tumors and their frequent location in critical brain areas that control speech, motor function, and the senses. Furthermore, the surgeon’s assessment of total tumor excision aligns with MRI-enhanced clearing in merely 30% of instances [7]. This mismatch may result in an inflated count of total resection cases in this study, thus diminishing the perceived influence of resection extent on survival outcomes.

Radiotherapy is the principal treatment approach for unresectable GBM. Radiotherapy is typically administered in conjunction with chemotherapy after surgery, utilizing various sequential combinations. The combination of radiotherapy and TMZ yields superior survival outcomes compared to radiotherapy alone in the treatment of GBM. TMZ is associated with unwanted systemic toxicity; therefore, combination strategies aimed at minimizing adverse effects while enhancing anti-tumor responses are critically needed [5,8,9].

Several FDA-approved drugs and one medical device are available for glioma management in addition to TMZ: lomustine, intravenous carmustine, carmustine wafer implants, bevacizumab (VEGFR inhibitor), and tumor treatment fields. The approved drugs and devices are primarily used in the management of recurrent high-grade gliomas, with only temozolomide, carmustine wafer implants, and tumor treatment fields being applicable for de novo diagnoses. With the exception of bevacizumab, FDA-approved medications for gliomas belong to the category of DNA-alkylating agents. Their mechanism of action is not specifically targeted at tumor cells, resulting in associated systemic adverse effects [10,11].

The combination of lomustine and TMZ chemotherapy demonstrated a significant improvement in overall survival compared to standard adjuvant therapy in patients with newly diagnosed GBM harboring a methylated MGMT promoter. This finding offers new evidence suggesting that dual-agent treatment may be more effective than TMZ monotherapy for GBM [6,12].

Over the past few years, significant clinical advancements have resulted from immunotherapy, which uses the body’s immune system to combat cancer. Numerous immunotherapy agents, including monoclonal antibodies targeting cytotoxic T-lymphocyte-associated protein 4 (CTLA-4), programmed cell death protein 1 (PD-1), and PD-1 ligand 1 (PD-L1), as well as CAR T cell therapy, have received approval from the U.S. Food and Drug Administration (FDA) for cancer treatment. Currently, there are no FDA-approved immunotherapies for GBM, and molecular heterogeneity in GBM is recognized as a significant driver of treatment resistance, posing a critical clinical issue in the development of effective immunotherapies targeting GBM [13].

Currently, oncolytic virotherapy constitutes a promising modality of immunotherapy for the treatment of GBM. There are two types of virotherapy: one in which replication-competent oncolytic viruses selectively infect and proliferate within cancer cells to induce tumor cell death, and another in which replication-deficient viral vectors are employed as vehicles for the delivery of therapeutic genes. To promote effective and selective replication, several OVs have been genetically modified to target pathogen-associated receptors found on tumor cells [14].

RTKs have recently become crucial biological targets in the pursuit of more effective treatments for GBM. RTKs, including epidermal growth factor receptor (EGFR), platelet-derived growth factor receptor (PDGFR), vascular endothelial growth factor receptor (VEGFR), epidermal growth factor, latrophilin, and seven transmembrane domain–containing protein on chromosome 1 (ELTD1), are essential in regulating cell growth, survival, and angiogenesis [15].

Targeting these receptors with tyrosine kinase inhibitors (TKIs) has shown promise, particularly with agents such as regorafenib, which significantly improved overall survival in recurrent GBM patients in the REGOMA trial [16]. However, Mongiardi et al. highlighted that many patients exhibit resistance to regorafenib and may experience significant side effects [17]. In particular, glioblastomas with alterations in the MAPK pathway respond poorly, while EGFRvIII-altered tumors show a better response [18]. This variability emphasizes the urgent need for identifying key molecular markers of regorafenib responsiveness across this heterogeneous cancer [17]. Meanwhile, Muñoz-Mármol et al. emphasized regorafenib’s difficulty in penetrating the blood–brain barrier, limiting its ability to reach tumor cells effectively [19]. These challenges contribute to the mixed clinical outcomes observed.

Other TKIs under investigation include sorafenib, which inhibits tumor growth and angiogenesis, and cediranib, a potent VEGFR inhibitor that has undergone clinical trials for GBM [20]. Additionally, combinations of TKIs, such as gefitinib and sunitinib, are being explored to target multiple signaling pathways and overcome resistance mechanisms [15,21,22,23,24].

The emergence of -omics technologies, such as transcriptomics, proteomics, and metabolomics, presents a significant opportunity to elucidate the complex molecular landscape of GBM. These methodologies facilitate the identification of critical genetic modifications, transcriptome profiles, protein interactions, and metabolic requirements associated with RTK dysregulation. Integrating -omics data enables researchers to achieve a comprehensive understanding of RTK signaling patterns and their contribution to tumor growth, discover novel biomarkers for diagnosis and prognosis, and pinpoint new therapeutic targets [25,26].

This paper examines the essential role of -omics technologies in elucidating RTK-driven pathways in GBM and emphasizes their potential to guide precision medicine approaches in the battle against this severe tumor.

## 2. RTK Signaling Pathways in GBM

### 2.1. Epithelial Growth Factor Receptor (EGFR)

EGFR is a transmembrane tyrosine kinase that is part of the erythroblastic leukemia viral oncogene homologue (ErbB) family of RTKs. Activation of EGFR in cancer cells enhances proliferation and safeguards altered cells against apoptosis. EGFR amplifications and mutations are identified in 40–60% of GBM multiforme cases. The prevalent mutational variation, EGFRvIII, is present in approximately 50% of individuals exhibiting EGFR amplification and results in constitutive activation of EGFR (Table 1) [27].

Activation of EGFR in GBM promotes tumor development via multiple critical downstream pathways. The PI3K/AKT/mTOR system facilitates cellular survival, proliferation, and metabolic adaptability, hence contributing to treatment resistance. The RAS/RAF/MEK/ERK pathway promotes proliferation, invasion, and matrix remodeling, whereas the JAK/STAT pathway governs the transcription of genes associated with survival, angiogenesis, and immune evasion.

The PLCγ/PKC pathway affects cytoskeletal dynamics and cellular motility, while SRC family kinases augment oncogenic signaling and increase invasiveness and angiogenesis. Furthermore, the NF-κB pathway facilitates inflammation, cellular survival, and resistance to therapy. The significant interaction and redundancy among these pathways enhance GBM’s plasticity, complicating treatment targeting. These observations underscore the necessity for multi-pathway inhibition techniques to effectively impede EGFR-driven carcinogenesis.

We will examine these pathways in greater detail in the subsequent sections, delineating their specific contributions to GBM proliferation, invasion, angiogenesis, and resistance mechanisms, as well as their implications for targeted therapeutic strategies.

In the field of therapeutic intervention, EGFR is nevertheless the most advantageous target. EGFR-targeted therapies are readily available; nonetheless, GBM frequently acquires resistance through the aforementioned mechanisms. These techniques include compensatory activation of alternate signaling pathways, mutations in EGFR, and alterations in receptor trafficking and degradation. We will further discuss these matters in a separate paragraph.

Given the intricate involvement of EGFR in multiple signaling pathways, its pivotal role in tumor growth, survival, and resistance mechanisms, and its frequent amplification and mutation in GBM, EGFR stands as a crucial target for future research and therapeutic interventions. Addressing EGFR’s complex network of interactions offers the potential for significant advancements in the treatment of GBM, making it an indispensable focus of scientific inquiry.

### 2.2. Platelet-Derived Growth Factor Receptor (PDGFR)

GBM cells are distinguished by the presence of PDGFRs, specifically PDGFRα and PDGFRβ, which are overexpressed and persistently active in this type of tumor. Overexpression of these molecules has been directly linked to the aggressive nature of GBM (Table 1) [27].

GBM cells produce PDGF ligands, including PDGF-A, PDGF-B, PDGF-C, and PDGF-D. These ligands engage with PDGFRs on adjacent cells (paracrine) or on the same cell (autocrine). This binding initiates downstream signaling pathways, similar to the ones observed with EGFR, that promote cell proliferation, survival, and migration [34].

Amplification of PDGFRα is a characteristic feature of the proneural subtype of GBM, playing a crucial role in cell proliferation and tumor cell signaling. Research demonstrates that PDGFRα signaling is crucial for glioma-like hyperplasia and the preservation of glioma stem cell characteristics via pathways such as PDGFRα/STAT3/RB1 [35]. Persistent autocrine activation of PDGFRα by PDGF-AA, in conjunction with the loss of p53, is essential for the activation of malignant cell proliferation [36].

The fast proliferation of GBM requires a substantial blood supply, which is achieved by mechanisms like vascular co-option and tumor angiogenesis. PDGFRβ signaling is essential in tumor angiogenesis, as genetic studies have detected PDGFRB expression in hyperplastic blood vessels and tumor microvasculature [29]. GBM expresses all PDGF ligands, facilitating tumor proliferation via autocrine and paracrine pathways. Glioma stem cells are capable of producing tumor pericytes, thereby modifying the microenvironment to facilitate growth. Tumor pericytes, characterized by markers such as αSMA and desmin, may facilitate immunosuppression in GBM [37].

Vascular mimicry, in which tumor cells assume endothelial cell–like characteristics, sustains tumor vasculature independent of angiogenesis. PDGF-BB–activated pericytes can attract macrophages, facilitating immune evasion [38]. GBM-associated stromal cells (GASCs), which express fibroblast markers such as αSMA and PDGFRβ, facilitate cancer invasion and metastasis [39]. PDGF-CC, which plays a role in blood vessel formation, is associated with VEGF production and stabilizes tumor vasculature, hence imparting resistance to anti-VEGF therapy [40].

PDGFR’s critical role in GBM underscores the necessity for continued research. Tumor heterogeneity presents significant challenges in the effective application of inhibitors, highlighting our incomplete understanding of PDGFR signaling. Given these complexities, further research is essential to unravel the intricacies of PDGFR pathways and develop more effective therapeutic strategies for combating GBM.

### 2.3. Vascular Endothelial Growth Factor Receptor (VEGFR)

VEGFRs are quintessential receptor tyrosine kinases (RTKs) including extracellular, transmembrane, and intracellular components, with the intracellular domain housing a tyrosine kinase domain. VEGFRs are classified into three categories, each with distinct ligand affinities: VEGFR1 interacts with VEGFA, VEGFB, and PIGF; VEGFR2 interacts with VEGFA, VEGFE, VEGFC, and VEGFD; and VEGFR3 interacts with VEGFC and VEGFD [41].

VEGFR1 and VEGFR2 are mainly implicated in vasculogenesis and angiogenesis, whereas VEGFR3 is associated with lymphangiogenesis. Ligand interaction with VEGFR induces receptor dimerization, activates the kinase, and leads to tyrosine phosphorylation, thereby activating signaling pathways that enhance cell proliferation, migration, and vascular tube formation [42].

Angiogenesis in GBM may be influenced by mechanisms associated with or independent of hypoxia. Hypoxia stabilizes hypoxia-inducible factor-1α (HIF-1α), which activates the VEGF gene, whereas GBM exhibit dysregulations in the Ras/MAPK and PI3K/Akt pathways, leading to upregulation of VEGF and other proangiogenic factors. This dual regulation highlights the intricacy of angiogenic signaling in GBM and the crucial function of VEGFRs in facilitating tumor growth and survival [43].

A direct correlation between overexpression of VEGFR and GBM development has been observed. VEGFR1 was identified on endothelial cells in 80% of recurrent GBM patients, whereas VEGFR2, which is generally missing in healthy tissues, was detected in 60% of samples. Both receptors, especially VEGFR2, exhibited elevated levels in GBM relative to lower-grade gliomas, and their expression correlated with glioma grade [30].

Significantly, VEGFR3, which is often absent in the brain, was also detected in GBM tissues [44]. The concentration of these receptors was greater in the tumor core, with VEGFR1 and VEGFR2 exhibiting increased expression in the tumor periphery relative to normal brain tissue. Constitutive phosphorylation of the tyrosine residues of VEGFR2 was detected in 71% of GBM specimens, but not in lower-grade gliomas (Table 2) [30].

Moreover, VEGFR1 and VEGFR2 exhibited heightened expression in tumor-educated platelets from GBM patients, suggesting their potential as indicators for treatment efficacy and disease advancement. VEGFR2 was associated with diminished progression-free survival in patients with relapsed high-grade glioma [30].

VEGFA, which disrupts the pericytes covering blood vessels, was consistently detected in GBM specimens, characterized by the greatest vessel diameters and elevated microvascular density [64]. VEGF expression was markedly elevated in GBM relative to lower-grade gliomas, associated with astrocytoma advancement and exhibiting a greater concentration at both the tumor center and periphery compared to normal brain tissue [65].

Soluble VEGFR1, an angiogenesis inhibitor, was elevated in GBM; nevertheless, VEGFA levels were sufficiently high to diminish the soluble VEGFR1-to-VEGFA ratio, signifying enhanced VEGFA activation in GBM. The VEGF-to–soluble VEGFR1 ratio has been identified as a significant predictive indicator, in conjunction with VEGF concentration and vascular surface area, in patients with malignant gliomas. Elevated levels of circulating endothelial progenitor cells in GBM patients were associated with enhanced vascular density, indicating their potential as biomarkers for identifying patients who may benefit from anti-angiogenic therapy [66].

These results emphasize the necessity of advancing our understanding of VEGFR signaling pathways to devise more effective anti-angiogenic therapies. By targeting VEGF and its receptors, there is potential to significantly impede tumor growth, improve patient outcomes, and overcome the limitations of current therapeutic approaches. Consequently, VEGF represents a crucial target for ongoing and future research, aiming to unveil new therapeutic avenues and enhance the efficacy of GBM treatment modalities.

### 2.4. c-MET and the Hepatocyte Growth Factor (HGF) Pathway

The MET proto-oncogene, located on chromosome 7q31, and HGF, located on chromosome 7q21.1, are crucial in glioma cell biology, affecting tumor proliferation, growth, migration, invasion, angiogenesis, and stemness. Approximately 30% of GBMs demonstrate overexpression of HGF and MET (Table 1), with heightened MET levels correlating with elevated WHO grades and diminished progression-free survival (PFS) and overall survival (OS) in patients. Immunohistochemical labeling revealed the presence of MET in tumor cells, blood vessels, and peri-necrotic areas of glioma specimens [31].

Analysis of GBM using TCGA data identified substantial genomic changes, particularly in genes located around 7q31–34, in addition to MET amplification. MET gain occurs in over 50% of primary and secondary GBMs, demonstrating its role in pathogenesis (Table 2). Activating mutations in MET are essential in the advancement of low-grade gliomas to secondary GBM, with MET amplification associated with reduced overall survival rates [67].

MET amplification and mutations are critical oncogenic occurrences in GBM, identified in 4% of clinical specimens (Table 1), leading to overexpression and persistent activation. Recent discoveries have found new METΔ7–8 mutations and fusion transcripts such as PTPRZ1-MET, which augment MAPK signaling and contribute to the progression of aggressive cancers (Table 2). The identification of MET amplification is dependent upon the techniques applied, such as FISH and CGH array, together with the particular antibodies used in IHC staining [68].

HGF, released by neurons and blood vessels, facilitates glioma invasion and the chemotactic migration of MET-positive cells, while also acting as a chemokine for microglial infiltration in malignant gliomas. These processes facilitate GBM progression, highlighting the disease’s aggressive nature [69].

The involvement of MET in GBM, marked by overexpression, amplification, and activating alterations, underscores its importance in tumor biology. The inconsistency in detecting techniques underscores the need for standardized methodologies. The intricate interactions between MET and HGF complicate the tumor microenvironment, establishing MET as a vital target for therapeutic intervention in GBM. Continued study is crucial to clarify the complexities of MET signaling and develop viable treatment strategies.

### 2.5. AXL Receptor

AXL and GAS6 overexpression in GBM patients is associated with poor outcomes and malignant aggressiveness. By encouraging actin rearrangement and micropinocytosis, phospho-AXL activation helps GBM cells penetrate and spread. In glutamine-rich environments, AXL-mediated micropinocytosis enhances GBM cell albumin absorption and proliferation. GBM cell invasion requires the GAS6-AXL signaling pathway, which involves PI3K. Cancers with active GAS6-AXL pathways may benefit from PI3K or AXL inhibitors to reduce metastasis [70].

As GBM is critical, new treatments are needed to increase survival and quality of life. Research indicates that αCTLA-4 therapy enhances survival in advanced GBM, despite limited immune checkpoint inhibitor effectiveness, necessitating CD4+ T cells. AXL/MER RTK signaling between CD4+ T cells and microglia enhances tumor suppression through IFNγ-dependent activation and phagocytosis. MHC-II molecules in microglia and dendritic cells are necessary for the CD4+ T cell response and tumor suppression, regardless of tumor cell MHC-II expression [32].

Quercetin and corosolic acid may suppress AXL to treat GBM. Quercetin, a bioactive flavonoid, kills GBM cells by inhibiting the AXL/IL-6/STAT3 signaling pathway, without affecting Akt or MAPK. This technique phosphorylates STAT3 and lowers IL-6. Corosolic acid stabilizes the cytoskeleton, decreases AXL and GAS6, and blocks GBM cell invasion. This is done by inhibiting JAK2, MEK, and ERK phosphorylation and F-actin expression [71].

AXL receptors confer GBM chemotherapy and radiation resistance. Research links recurrent TMZ delivery in hypoxic circumstances to increased CT-AXL levels, associated with HIF1α and treatment resistance. Traditional therapy with R428 (bemcentinib) improves efficacy and manages resistance. Targeting AXL improves GBM treatment [32].

### 2.6. RTK Downstream Signaling Pathways

**Figure 1:**
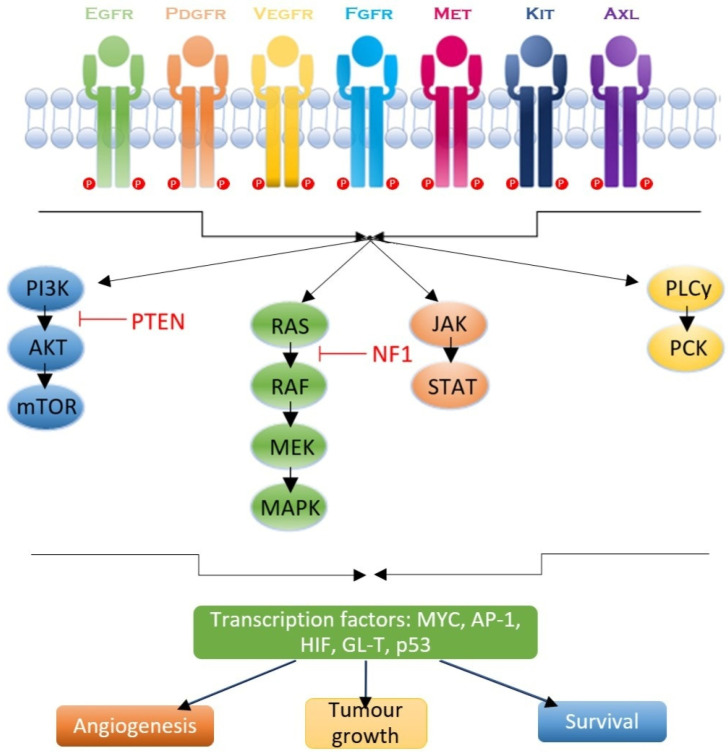
RTK signaling pathways and downstream effects in glioblastoma.

Following ligand interaction, the previous receptors can further activate complex signaling pathways, which we will thoroughly describe in this section. The pathways encompass many pathways, of which the most important are the PI3K/protein kinase B (PKB)/AKT pathway, the RAS/mitogen-activated protein kinase (MAPK)/extracellular signal–regulated kinase (ERK) pathway, the Janus kinase (JAK)/STAT pathway, and the phospholipase C (PLC)/protein kinase C (PKC) pathway. These pathways facilitate cellular proliferation, survival, angiogenesis, and invasion of other sites [72]. A comprehensive overview of the multi-omics characteristics—including mutation frequencies, functional consequences, and recent findings—of these key RTKs is provided in Figure 1.

#### 2.6.1. RAS/MAPK/ERK Pathway

The RAS/MAPK/ERK pathway encompasses small GTPases such as RAS proteins, which are modulated by GTPase-activating proteins and guanine nucleotide exchange factors (GEFs). EGFR activation exhibits RAS action, which in turn initiates the RAF-MEK-ERK1/2 signaling cascade, resulting in phosphorylation of ERK1/2, which has been proven to directly influence cell proliferation, survival, and metabolism [73]. While RAS mutations are infrequent in GBM (2%), elevated RAS activity is noted, accompanied by NF1 mutations or deletions in 18% of patients [33]. These modifications underscore the significance of the EGFR/RAS/MEK/ERK pathway in the etiology of GBM [74].

#### 2.6.2. JAK/STAT Pathway

JAKs are RTKs that interact with cytokine receptors. The interaction between JAK2 and EGFR results in resistance to EGFR inhibitors. Following cytokine binding, JAK activates and phosphorylates STAT proteins, which dimerize and translocate to the nucleus to modulate the transcription of genes associated with transformation, cancer, stemness, and migration [75]. STAT3 can be directly phosphorylated by EGFR, resulting in its dimerization. The function of STAT3 in GBM multiforme carcinogenesis is directly influenced by other gene alterations [76]. Although STAT3 normally inhibits astrocyte transformation mediated by phosphatase and tensin homolog (PTEN) loss, when associated with the active form—EGFRvIII—it promotes malignant transformation. Activation of STAT3 in this case will facilitate tumor proliferation by suppressing immunological responses and enhancing stemness and angiogenesis [75].

#### 2.6.3. PI3K/AKT Pathway

PI3Ks are enzymes that phosphorylate cellular lipids and are categorized into three classes according to their structural characteristics and substrate selectivity. Class IA PI3Ks, comprising catalytic p110 and regulatory p85 subunits, are pivotal in oncogenesis. Active EGFR interacts with p85, mitigating its inhibitory influence and allowing p110 to phosphorylate PIP2 into PIP3. This establishes a docking site for AKT, which is partially activated by PDK1 and fully activated by mTORC2. PTEN, a tumor suppressor, catalyzes the dephosphorylation of PIP3 to PIP2, hence inhibiting PI3K/AKT signaling. Deletion of chromosome 10q, encompassing PTEN, occurs prior to EGFR amplification in GBM multiforme [77].

#### 2.6.4. PLC/PKC Pathway

Active EGFR binds and activates PLC, which hydrolyzes PIP2 into inositol 1,4,5-trisphosphate and diacylglycerol. Activated PLC subsequently activates PKC, a substantial family of serine/threonine kinases [78]. Protein kinase C (PKC) isoforms, categorized into classic, nonclassic, and atypical families, operate as either tumor suppressors or oncogenes contingent upon the contextual environment [79]. They modulate tumor proliferation, angiogenesis, infiltration, and survival by activating effectors such as p53, p21, RAS-RAF1, and many others. EGFR transmits signals to mTOR in a way reliant on PKC. Inhibition of PKC reduces the viability of GBM cells, underscoring the essential function of PKC in GBM [80].

## 3. Recent Advances in RTK -Omics Approaches and Their Impact on Diagnosis and Therapeutic Targets in *GBM*

### 3.1. Genomics

Genomic studies, particularly through resources like The Cancer Genome Atlas (TCGA), have provided crucial insights into the molecular landscape of GBM. TCGA data reveal frequent mutations, amplifications, and alterations in key signaling pathways that drive the aggressiveness of this cancer. A major contributor to GBM progression is the dysregulation of RTKs, which regulate critical cellular processes such as growth, survival, and migration. Alterations in RTKs, including EGFR amplification and PDGFR mutations, are commonly observed in GBM and lead to aberrant activation of downstream signaling pathways. This subsection delves into the genomic characterization of RTK alterations in GBM, highlighting their role in tumorigenesis and their potential as targets for therapeutic intervention.

The TCGA GBM dataset elucidates a specific group of tumors distinguished by EGFR amplification frequently associated with TP53 mutations. The observed alterations demonstrate a significant degree of mutual exclusivity, indicating a sophisticated regulatory interplay in which EGFR diminishes the activity of wild-type p53. Moreover, activating mutations in PIK3CA, which are associated with approximately 15% of GBM cases, contribute to tumor recurrence and unfavorable outcomes through dysregulation of the PI3K signaling pathway. Analyses of genomic data has yielded essential insights into oncogenic events, identifying targets such as EGFR and PIK3CA that are fundamental to the pathophysiology of and treatment approaches for GBM [28].

The TCGA consortium has significantly enhanced our comprehension of molecular changes in GBM by supplying extensive genomic data from substantial patient cohorts. The TCGA has identified many MET abnormalities, including localized amplification, gene fusions, and exon 14 skipping, which are pivotal in GBM pathogenesis and affect tumor growth and clinical outcomes. Focal MET amplification results in persistent kinase activity and unfavorable prognosis, although MET inhibitors exhibit potential in combination therapy. MET gene fusions, including TPR-MET and PTPRZ1-MET, lead to persistent activation of oncogenic signaling pathways such as MAPK, which contributes to aggressive tumor characteristics and treatment resistance. MET exon 14 skipping, which hinders receptor degradation, promotes persistent MET activation and adverse prognosis. Targeted treatments, such as MET inhibitors like crizotinib and cabozantinib, are under investigation, demonstrating some efficacy in preclinical and clinical research; nevertheless, resistance continues to be a hurdle [57].

Genomic investigation of GBM indicates a significant prevalence of sequence changes, with 28.6% of mutations located in hotspot regions. Significantly, GBM exhibits a higher prevalence of subclonal mutations in these hotspots (7.0%) relative to malignancies such as breast and lung cancers and melanoma. In IDH1 wild-type high-grade gliomas, targeted therapies corresponding to actionable mutations, including BRAF, NF1, MET, and PDGFRA amplifications, have demonstrated encouraging outcomes. Trametinib and dabrafenib for BRAF mutations, along with cabozantinib for MET amplifications, resulted in partial responses and extended progression-free survival (PFS) and overall survival (OS), underscoring the significance of genetics in customizing successful therapies for GBM [53].

In conclusion, genomic investigations, particularly via TCGA, have identified critical molecular drivers of GBM, such as mutations in EGFR, PDGFR, MET, and PIK3CA. These modifications facilitate tumor progression and therapeutic resistance. Although targeted medicines demonstrate promise, obstacles such as resistance and the intricacies of subclonal mutations persist. Continued research and individualized treatment approaches are essential for enhancing GBM outcomes.

### 3.2. Transcriptomics

Transcriptomics, which thoroughly investigates gene expression at the transcript level, is essential for comprehending the molecular underpinnings of GBM. Transcriptomic approaches provide essential insights into the mechanisms of tumor growth and resistance by simultaneously monitoring the activity of hundreds of genes and facilitating the identification of biomarkers and therapeutic targets. Recent advancements in transcriptomic methodologies, including microarray analysis, RNA sequencing (RNA-seq), and single-cell RNA sequencing (scRNA-seq), have significantly improved the scope and accuracy of gene expression research in GBM [81].

Microarray analysis has been extensively employed in GBM research to contrast tumor specimens with normal cerebral tissue. This method facilitates the identification of differentially expressed genes (DEGs) that play a role in tumor growth, including VEGF, which is implicated in angiogenesis. Nonetheless, its constraints, such as diminished sensitivity and reliance on pre-fabricated probes, have prompted the use of more sophisticated methodologies like RNA-sequencing (RNA-seq) [82].

RNA-seq is favored because of its superior sensitivity and resolution relative to microarrays, facilitating the identification of novel transcripts, gene fusions, and unusual isoforms. It has been essential in identifying oncogenic fusion genes such as FGFR-TACC and alternative splicing events in GBM [83].

Single-cell RNA sequencing (scRNA-seq) facilitates the analysis of tumor heterogeneity at single-cell resolution, uncovering unique subpopulations within the GBM tumor microenvironment. This method has detected transcriptional differences across GBM stem-like cells, differentiated tumor cells, and immune cells, revealing the intricacies of therapeutic resistance [84].

A study conducted by Xu et al. (2024) [85] focused on transcriptomics and proteomics in relation to glioma growth. The researchers analyzed the functions of TIMP1 and CHI3L1 to explore the molecular pathways and alterations in gene expression associated with the aggressive proliferation and immune evasion exhibited by glioma. This study indicated that activation of the NF-κB pathway reflects significant interplay between proteins and transcripts that facilitates tumor development and immunosuppression. The study incorporated various -omics approaches to thoroughly elucidate the underlying biological pathways [85].

Transcriptomic analysis offers enhanced insight into the transcriptional ramifications of mutations in GBM. Activation of EGFR, for instance, induces transcriptome alterations that facilitate tumor proliferation and resistance mechanisms. Likewise, disruption of the PI3K pathway, especially with PIK3CA mutations, modifies transcriptional networks critical for cellular survival and metabolic flexibility. Furthermore, transcriptome profiling facilitates the identification of expression patterns of critical factors such as PTK2, whose increased mRNA levels in GBM promote greater adhesion, migration, and survival. By clarifying these transcriptional alterations, transcriptomics improves our understanding of how genomic anomalies result in functional cellular effects, hence guiding therapeutic advancement [86].

A transcriptomic analysis study published in late 2024 utilized single-cell RNA sequencing (scRNA-seq) of glioma data, facilitating intricate network analysis across various cancer stages. The study identified essential ligand–receptor interactions and a significant ligand–receptor–transcription factor (TF) axis, along with corresponding biological pathways. Differential network analysis of grade III and grade IV gliomas revealed essential nodes and interactions, with pathway enrichment emphasizing four pivotal genes—PDGFA, PDGFRA, CREB1, and PLAT—linked to the RTK signaling pathway, which is crucial for glioma progression. These genes served as features in machine learning models, attaining 87% accuracy and 93% AUC in forecasting glioma progression and 3-year survival, offering significant insights for prognosis and treatment approaches [87].

Transcriptomic innovations, notably bulk RNA-seq and scRNA-seq, have transformed comprehension of GBM’s molecular mechanisms, especially in the context of RTKs. These techniques offer significant insights into changes in gene expression that promote cancer and create new opportunities for treatment. Future investigations integrating transcriptomics with multi-omics methodologies are expected to enhance treatment alternatives for this aggressive malignancy.

### 3.3. Proteomics

Proteomics plays a crucial role in understanding GBM by revealing protein expression and post-translational changes and identifying biomarkers and therapeutic targets. Mass spectrometry, commonly used in GBM research, enables sensitive protein quantification, highlighting modifications like EGFR overexpression and PTEN downregulation, both key to tumor growth and resistance. Recent studies have identified oncogenic fusion proteins and altered signaling pathways, offering potential treatment targets [63].

Proteomics facilitates the comprehension of protein–protein interactions (PPIs) and protein localization in GBM. Recent breakthroughs in affinity purification mass spectrometry (AP-MS) and co-immunoprecipitation (Co-IP) have delineated protein complexes implicated in cellular survival and resistance, with pathways such as PI3K/AKT being pivotal to GBM progression [88]. Modified protein location also facilitates the tumor’s capacity to dodge apoptosis, a critical aspect of its treatment resistance.

Two-dimensional gel electrophoresis (2-DE) and protein microarrays have been employed to examine protein alterations in response to treatment, elucidating resistance mechanisms, particularly those associated with TMZ therapy [89]. Despite their reduced throughput, these approaches have been crucial in finding biomarkers associated with unfavorable prognosis.

EGFR signaling, for example, regulates the activity of proteins like DNA-PKcs, diminishing wild-type p53 function via protein–protein interactions. Proteomic investigations reveal the significance of PTK2, as its increased protein levels and phosphorylation states correlate with GBM advancement. Moreover, the ubiquitin–proteasome system (UPS), which regulates protein stability and degradation, serves as a crucial modulator of signaling networks, including those associated with EGFR and p53. Proteomics has identified these alterations and facilitated the examination of post-translational modifications, thereby augmenting our comprehension of protein function and treatment susceptibility in GBM [90].

Recent advancements in bioinformatics and multi-omics integration are mitigating obstacles in data interpretation and protocol standardization. The integration of proteomics with genomes and transcriptomics offers a more thorough understanding of the molecular landscape of GBM, revealing novel treatment targets and enhancing prognostic accuracy.

### 3.4. Metabolomics

Metabolomics has become an important instrument in exploring the intricate metabolic reprogramming in GBM, especially as it uncovers the tumor transition from oxidative phosphorylation to glycolysis (Warburg effect). Researchers have emphasized the significant influence of the tumor microenvironment, encompassing hypoxia and nutritional deficiencies, in inducing metabolic changes that differ among GBM subtypes. This has resulted in the identification of distinctive metabolic signatures that can facilitate more accurate tumor classification [91].

Neoplasms exhibiting elevated glycolytic activity are generally more aggressive, rendering lactate and glycolytic intermediates significant indicators. Recent studies indicates that malignancies characterized by increased glutaminolysis and lipid metabolism display unique metabolic dependencies, presenting new opportunities for targeted therapeutics focused on these pathways [92].

Metabolomic profiling has demonstrated efficacy in subclassifying GBM and forecasting tumor behavior. Non-invasive methods, like magnetic resonance spectroscopy (MRS), are very significant for identifying distinct metabolic signatures, including increased lactate and choline, which facilitate GBM categorization and real-time assessment of therapy efficacy [93].

Recent research on the metabolic reprogramming of cancer cells, especially in GBM multiforme, underscores notable relationships among tumor mutations, metabolic profiles, and microenvironments. Metabolomic and lipidomic analyses have identified critical metabolites such as choline (Cho), phosphocholine (PC), glycerophosphocholine (GPC), glutamine (Gln), glutamate (Glu), γ-aminobutyric acid (GABA), myo-inositol, and 2-hydroxyglutarate (2-HG) as prospective biomarkers for the diagnosis, grading, and prognostication of GBM [93].

Altered concentrations of Cho and its derivatives, including PC and GPC, are associated with particular GBM mutations (e.g., PDGFRA, EGFR), with elevated PC levels signifying high-grade gliomas [94].

Myo-inositol overexpression and glutamine reliance correlate with more aggressive forms of GBM multiforme, although reduced glutathione levels indicate a poorer prognosis. IDH1 mutations result in increased 2-HG levels, aiding in the differentiation of low- and high-grade gliomas [95].

Metabolomics provides a distinct perspective on the metabolic reprogramming induced by oncogenic mutations in GBM multiforme. The PI3K signaling pathway, frequently initiated by PIK3CA mutations, regulates substantial alterations in cellular metabolism, augmenting energy production and metabolic activities essential for tumor proliferation. Likewise, EGFR activation affects metabolic adaptation by regulating essential enzymes and pathways. Metabolomics elucidates the impact of the ubiquitin–proteasome system on metabolic homeostasis, with research indicating that inhibition of ubiquitin signaling counteracts metabolic reprogramming in GBM. Metabolomics has elucidated metabolic alterations, offering insights into the biochemical ramifications of genetic and proteomic modifications, hence facilitating the development of innovative metabolic-targeted therapeutics for GBM treatment [90].

A 2024 study by Fontanilles et al. aimed to examine metabolic remodeling in GBM, with the observed alterations in metabolomic profiles offering insights into this dynamic process. RTK activation, especially via the EGF/EGFR pathway, induces substantial metabolic reprogramming, as evidenced by alterations in glycerophospholipids (e.g., PC ae C42:4 and PC ae C42:5) and acylcarnitines, which play roles in membrane composition and cellular energy consumption. These metabolic alterations are probably induced by RTK signaling, affecting lipid metabolism and cellular mechanisms essential for tumor proliferation and treatment resistance. This study’s dynamic approach indicates that the observed correlations show a strong association between metabolic alterations and RTK activity, emphasizing the significance of metabolic remodeling in GBM growth [96].

These findings highlight the significance of metabolic indicators in the characterization and prognosis of GBM, stressing the necessity for analyzing extensive panels of metabolites and their ratios to enhance diagnostic and treatment strategies.

Nonetheless, obstacles persist, such as the intricacy of metabolomics data and the necessity for multi-omics integration. Moreover, empirical observation of clinical expression correlated with elevated biomarkers is needed to be able to thoroughly comprehend the mechanisms at hand. Thus, we will further address this necessary inter-play in the next section.

### 3.5. The Interplay of Multi-Omics Sciences and Clinical Data

**Figure 2:**
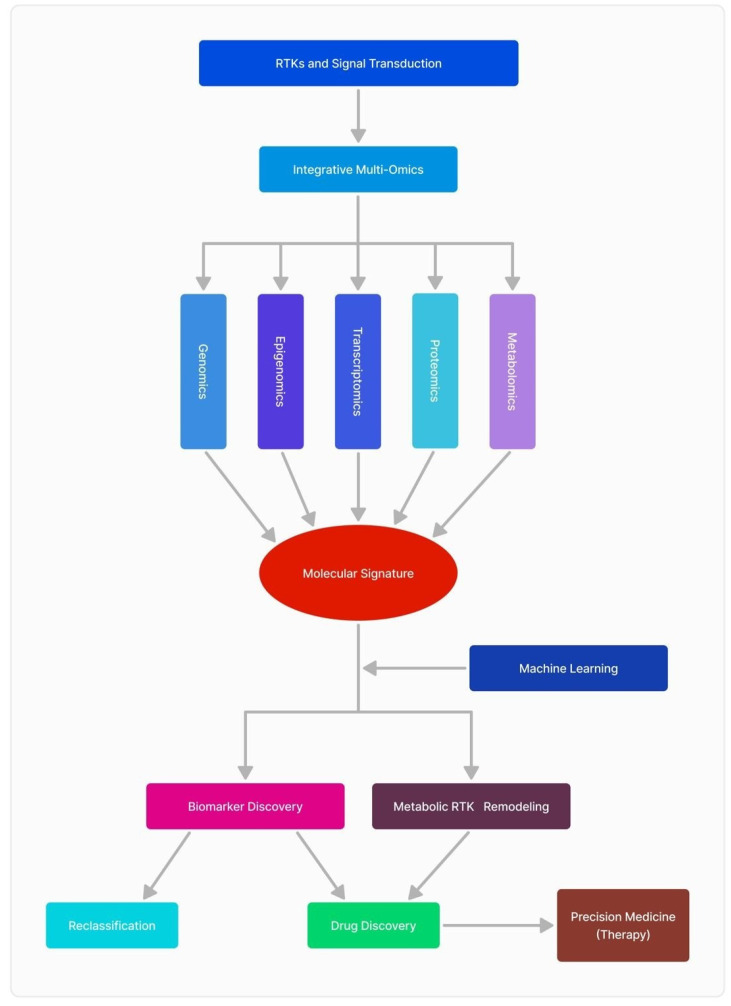
RTKs integrative multi-omics approaches in GBM research.

Through the integration of genomes, transcriptomics, proteomics, and metabolomics, researchers may construct a comprehensive framework of tumor biology, facilitating the discovery of novel biomarkers, therapeutic targets, and individualized treatment methods customized to each tumor’s own molecular profile. This method signifies a notable progression in personalized medicine, offering enhanced therapeutic results (Figure 2).

RNA sequencing has identified gene expression profiles that forecast improved survival rates in patients receiving immune checkpoint medications. The integration of genomic and transcriptome data has revealed a new GBM subtype, MES-IG, which exhibits a favorable response to immunotherapy, underscoring the efficacy of comprehensive molecular profiling [97]. Crucial proteins associated with adverse outcomes, such as PIM1, have been identified, uncovering novel treatment targets [98].

Single-cell RNA sequencing has revealed considerable variety within GBM tumors, indicating various cellular populations that may exhibit varying responses to treatments. This intratumoral diversity poses therapeutic obstacles while highlighting the significance of multi-omics in comprehending and tackling this complexity for enhanced treatment efficacy [99].

The use of multi-omics and clinical data augments our comprehension of GBM’s molecular underpinnings and possesses significant promise for clinical application. Biomarkers revealed via metabolomics, including elevated glycolytic activity, assist in tumor subclassification and therapy customization. Progress in non-invasive diagnostic technologies such as magnetic resonance spectroscopy (MRS), alongside multi-omics profiling, provides insights into tumor metabolism and therapeutic effectiveness [100].

A 2024 study by Liu et al. investigated the molecular differences between IDH-mutant astrocytomas and GBM multiforme using a sophisticated multi-omics methodology. DNA methylation analysis revealed substantial changes in CpG sites in IDH-mutant tumors, indicating dysregulated RTK signaling pathways. Proteomic and metabolomic analyses highlighted this dysregulation, with significant increases in protein (e.g., PDGFRA, PLCB1) and metabolite (e.g., 2-HG, glycerol 3-phosphate) levels in IDH-mutant tumors, indicating changes in cellular signaling and metabolism [101].

Transcriptomic research indicates diminished hypoxia signaling in IDH-mutant astrocytomas, characterized by decreased expression of HIF1A-associated genes and improved survival prognosis for patients exhibiting low hypoxia scores. This study integrated many -omics platforms to elucidate the molecular landscape of IDH-mutant cancers, emphasizing the role of epigenetic, protein, metabolic, and transcriptional alterations in tumor biology. This intricate, multi-faceted strategy underscores the significance of integrating all accessible resources to produce a more comprehensive and nuanced comprehension of GBM etiology [101].

Another 2024 study by Alom et al. researched how a new promising molecule, GMFG (glia maturation factor gamma), is upregulated in GBM, with its high expression correlating with poor overall survival (OS) in patients. Analysis of hub differentially expressed genes (DEGs) through GO and pathway enrichment revealed key processes and pathways involved in GBM, reinforcing GMFG’s potential as a diagnostic tool and therapeutic target. The study also demonstrated that GMFG’s elevated expression in GBM tissues suggests it may be used as a biomarker for GBM diagnosis and prognosis [102].

Research has shown that risperidone and 5′-guanidinonaltrindole showed significant docking energy when interacting with GMFG, suggesting their potential as therapeutic agents. Molecular dynamics (MD) simulations confirmed the stability of the interaction between the active pocket of GMFG and these compounds, with risperidone emerging as a potential target for GBM treatment. These findings contribute to a deeper understanding of GBM biology and offer promising avenues for drug development and disease management [102].

**Figure 3:**
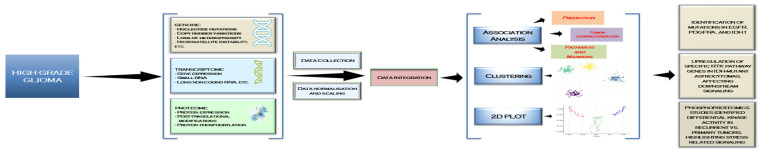
Multi-omics data integration for RTK signaling analysis in glioblastoma.

The integration and corroboration of data from various -omics platforms alongside clinical data present significant complexity and challenges. Each -omics layer offers a distinct viewpoint on tumor biology, operating at varying scales and employing diverse methodologies, including DNA sequencing, protein quantification, and metabolic profiling. This presents a complex challenge, as the relationships between molecular changes and clinical results are frequently not readily discernible. Additionally, variability among patients, heterogeneity of tumors, and the impact of external factors like treatment regimens complicate the process [100] (see Figure 3).

The process of integration begins with data collection from diverse sources, including genomic mutations (e.g., EGFR, PDGFRA, IDH1), transcriptomic alterations in RTK pathways, and proteomic insights into protein interactions and phosphorylation events. After normalization and scaling, data integration enabled association analyses, clustering, and visualization through 2D plots, highlighting critical RTK-driven mechanisms in GBM progression. These insights revealed key findings, such as methylation alterations near RTK-related genes affecting their expression, upregulation of specific RTK pathways influencing downstream signaling, and changes in chromatin accessibility driving tumor evolution. By leveraging multi-omics integration, researchers can pinpoint molecular vulnerabilities, paving the way for more effective therapeutic strategies targeting RTKs in GBM [101] (see Figure 3).

Translating these findings into clinically actionable knowledge requires the identification of significant biomarkers and their validation in diverse, real-world patient populations. The integration of these data types must consider biological complexity and clinical context; misinterpretation or oversight may result in flawed conclusions or missed therapeutic opportunities.

## 4. Conclusions

The thorough study of RTKs and their involvement in GBM pathophysiology has greatly enhanced our comprehension of this aggressive cancer. RTKs including EGFR, VEGFR, PDGFR, MET, and AXL regulate essential activities such as cell proliferation, survival, invasion, and angiogenesis, enhancing glioblastoma’s resistance to standard treatments. Notwithstanding these limitations, the incorporation of multi-omics methodologies—genomics, transcriptomics, proteomics, and metabolomics—has elucidated complex signaling networks, revealing prospective treatment targets and biomarkers.

Multi-omics research has identified distinct genomic mutations, transcriptome profiles, proteomic changes, and metabolic modifications caused by RTK dysregulation, highlighting their critical significance in tumor growth and therapeutic resistance. Innovations like single-cell transcriptomics and metabolic profiling have underscored intratumoral heterogeneity and metabolic interdependence, establishing a basis for precision treatment.

Recent studies have demonstrated that targeting RTK-driven pathways with multi-target tyrosine kinase inhibitors such as regorafenib has significantly improved survival outcomes in recurrent GBM patients (REGOMA trial) [16]. Furthermore, single-cell RNA sequencing (scRNA-seq) has provided crucial insights into the transcriptomic heterogeneity of glioblastomas, identifying key genes such as PDGFA, PDGFRA, CREB1, and PLAT that are strongly linked to tumor progression and patient survival [87]. These findings support the growing role of machine learning models in predicting glioma prognosis with high accuracy (87%) and AUC (93%), reinforcing the necessity of molecular profiling in guiding therapeutic strategies [87].

This study highlights the transformational potential of multi-omics analysis in GBM research, connecting molecular insights with therapeutic applications. Despite substantial obstacles, such as intratumoral heterogeneity and treatment resistance, ongoing investigation of RTK-driven pathways and their systemic consequences holds potential. The recent identification of GMFG as a novel biomarker and potential drug target further expands the therapeutic landscape, with studies showing that small molecules like risperidone demonstrate promising interactions with GMFG, suggesting a new avenue for GBM treatment [102]. Multi-omics techniques are pivotal in transforming the diagnosis, prognosis, and management of GBM by enhancing individualized treatment options and maximizing therapeutic outcomes, thereby providing hope to patients afflicted by this debilitating disease.

## Figures and Tables

**Table 1:** Recent insights into RTK mutations in GBM.

Receptor/RTK	Mutation Frequency	Functional Consequences	Recent Findings
EGFR	40–60% of GBM cases show amplification or mutation	Activates the PI3K/AKT, RAS/RAF/MEK, and JAK/STAT pathways, driving proliferation and therapy resistance	EGFR suppresses p53 by promoting DNA-PKcs binding, leading to resistance [28]
PDGFR	Amplifications in the proneural subtype of GBM	Promotes angiogenesis, tumor cell migration, and ECM remodeling	PDGFRβ+ pericytes attract macrophages, facilitating immune evasion [29]
VEGFR	VEGFR1 and VEGFR2 overexpressed in 60–80% of GBM samples	Drives hypoxia-induced vascular proliferation and therapy resistance	Tumor-educated platelets show increased VEGFR1 and VEGFR2 expression, correlating with disease progression [30]
MET	Amplifications in 30% of GBM, exon 14 skipping mutations	Enhances migration, invasion, and MAPK pathway activation	METΔ7–8 mutations and PTPRZ1-MET fusions increase MAPK signaling and drive aggressive phenotypes [31]
AXL	Overexpressed in GBM, correlating with poor prognosis	Enhances immune evasion, therapy resistance, and invasion	AXL activation in glutamine-rich environments increases micropinocytosis, aiding GBM survival [32]
NF1	Mutated in 18% of GBM cases	Loss of function mutations disrupt RAS regulation, leading to unchecked proliferation	NF1 mutations associated with aggressive GBM subtypes and poor prognosis [33].

**Table 2:** Multi-omics characterization of key RTKs in GBM.

RTK	Genomics	Transcriptomics	Proteomics	Metabolomics
EGFR	EGFR amplifications and EGFRvIII mutations drive tumor aggressiveness. PIK3CA mutations cause disruption in the PI3K pathway, contributing to recurrence [45].	Transcriptomic analysis identifies betacellulin (BTC) and epiregulin (EREG) as key regulators of EGFR in GBM, influencing its activation and mutation sensitivity, refining EGFR-targeted therapies [46].	EGFR overexpression and PTEN downregulation promote tumor growth and resistance. Phosphorylation (Y1068, Y1173) and PI3K/AKT signaling enhance cell survival and migration [47].	Activation of EGFR leads to reprogramming of lipid metabolism and glycolysis, enhancing energy production and tumor survival. Studies show elevated glycerophospholipids [48].
VEGFR	IDH1 R132H mutations in GBM are linked to increased HIF-1 alpha and VEGF levels, suggesting a role in tumor progression via hypoxia pathways [49].	SOCS3-VEGFA-TEK transcriptomic signature for GBM prognosis, linking SOCS3 expression to VEGFA-driven neovascularization and response to anti-angiogenic therapy [50].	VEGFR phosphorylation at key sites (Y951, Y1175) activates angiogenesis and cell survival pathways. Interactions with neuropilin enhance signaling [51].	VEGFR signaling promotes glycolysis, fatty acid oxidation, and mitochondrial biogenesis, supporting tumor survival under low-oxygen conditions [52].
PDGFR	PDGFR amplifications and mutations in the proneural subtype drive tumor progression by altering extracellular matrix (ECM) remodeling and promoting invasion [53].	PDGFR is enriched in the proneural subtype of GBM, affecting migration, adhesion, and immune evasion. Altered transcriptional networks support these processes [54].	PDGFR inhibition with JNJ disrupts phosphorylation, halting GBM growth via mitotic arrest and caspase-dependent apoptosis. Combined IGF-1R/PDGFR blockade enhances therapeutic efficacy [55].	Metabolic coupling between tumor and stromal cells promotes lactate production and aerobic glycolysis, supporting tumor invasiveness [56].
MET	MET amplifications, exon 14 skipping, and gene fusions (e.g., TPR-MET, PTPRZ1-MET) lead to persistent kinase activity and poor prognosis in GBM [57].	c-MET inhibition induces significant transcriptomic changes, including increased PGC1α expression regulated by cAMP response elements binding protein, driving oxidative metabolism in GBM [58].	c-MET inhibition drives mitochondrial fusion and reactive oxygen species production in GBM, revealing a shift in protein expression linked to oxidative metabolism [58].	c-MET inhibition induces metabolic reprogramming in GBM, enhancing oxidative phosphorylation and fatty acid oxidation, along with increased acyl-carnitines and anaplerosis [58].
AXL	AXL overexpression is associated with epithelial–mesenchymal transition (EMT), enhancing immune evasion and metastasis in GBM [32].	Transcriptomic analysis and shRNA screening identified AXL as a therapeutic target, highlighting its role in MES GSC survival in GBM [59].	Through protein expression analysis, P-AXL patterns in GBM were linked to survival, indicating its potential as a therapeutic target [60].	PROS1/AXL signaling in GSCs triggers metabolic reprogramming, including enhanced oxidative phosphorylation and fatty acid oxidation, supporting GBM growth [61].
HER2	HER2 overexpression is linked to therapy resistance and aggressive GBM phenotypes, contributing to tumor progression and poor prognosis [45].	HER2 gene expression changes induced by NK-92/5.28.z therapy, identifying immune response and tumor progression pathways [62].	Laser capture microdissection–based proteomic analysis improves HER2 detection in glioblastoma, ensuring accurate tumor-specific data for targeted therapy decisions [63].	HER2-driven metabolic changes in glycolysis, oxidative phosphorylation, and amino acid metabolism influenced by CAR NK cell therapy [62].
